# Effect of a vapor barrier in combination with active external rewarming for cold-stressed patients in a prehospital setting: a randomized, crossover field study

**DOI:** 10.1186/s13049-024-01204-2

**Published:** 2024-04-25

**Authors:** Sigurd Mydske, Guttorm Brattebø, Øyvind Østerås, Øystein Wiggen, Jörg Assmus, Øyvind Thomassen

**Affiliations:** 1 Department of Anaesthesia & Intensive Care, Haukeland University Hospital, Bergen, Norway; 2 Mountain Medicine Research Group, The Norwegian Air Ambulance Foundation, Bergen, Norway; 3 Department of Clinical Medicine, University of Bergen, Bergen, Norway; 4 Present Address: Norwegian National Advisory Unit on Emergency Medical Communication, Haukeland University Hospital, Bergen, Norway; 5 SINTEF Technology and Society, Preventive Health Research, Trondheim, Norway

**Keywords:** Accidental hypothermia, Treatment, Insulation method, Vapor barrier

## Abstract

**Background:**

Use of a vapor barrier in the prehospital care of cold-stressed or hypothermic patients aims to reduce evaporative heat loss and accelerate rewarming. The application of a vapor barrier is recommended in various guidelines, along with both insulating and wind/waterproof layers and an active external rewarming device; however, evidence of its effect is limited. This study aimed to investigate the effect of using a vapor barrier as the inner layer in the recommended “burrito” model for wrapping hypothermic patients in the field.

**Methods:**

In this, randomized, crossover field study, 16 healthy volunteers wearing wet clothing were subjected to a 30-minute cooling period in a snow chamber before being wrapped in a model including an active heating source either with (intervention) or without (control) a vapor barrier. The mean skin temperature, core temperature, and humidity in the model were measured, and the shivering intensity and thermal comfort were assessed using a subjective questionnaire. The mean skin temperature was the primary outcome, whereas humidity and thermal comfort were the secondary outcomes. Primary outcome data were analyzed using analysis of covariance (ANCOVA).

**Results:**

We found a higher mean skin temperature in the intervention group than in the control group after approximately 25 min (*p* < 0.05), and this difference persisted for the rest of the 60-minute study period. The largest difference in mean skin temperature was 0.93 °C after 60 min. Humidity levels outside the vapor barrier were significantly higher in the control group than in the intervention group after 5 min. There were no significant differences in subjective comfort. However, there was a consistent trend toward increased comfort in the intervention group compared with the control group.

**Conclusions:**

The use of a vapor barrier as the innermost layer in combination with an active external heat source leads to higher mean skin rewarming rates in patients wearing wet clothing who are at risk of accidental hypothermia.

**Trial registration:**

ClinicalTrials.gov identifier: NCT05779722.

**Supplementary Information:**

The online version contains supplementary material available at 10.1186/s13049-024-01204-2.

## Background

Accidental hypothermia is defined as an involuntary decrease in core body temperature below 35 °C, whereas the term “cold stress” refers to the body being subjected to a cold environment requiring a compensatory physiological response to avoid a decrease in core temperature [1, 2]. Hypothermia is a risk factor for cardiac arrythmia, pulmonary edema, coagulopathy, and neurological pathology [3, 4]. Isolated accidental hypothermia is potentially lethal on its own as well as being an independent risk factor for increased morbidity and mortality in patients with traumatic injury or other sources of hemorrhage [5, 6].

When a person is subjected to cold stress, the body automatically activates compensatory mechanisms to increase thermal production and minimize heat loss to maintain or restore a normal temperature. The mechanisms underlying thermal homeostasis are effective, complex, and closely maintained [1].

Evaporation of water from the skin is one of the four mechanisms of heat loss, along with radiative, convective, and conductive heat losses [7]. Evaporation plays a physiological role in core temperature homeostasis. When the temperature exceeds an upper threshold level, the body excretes sweat onto the skin, which cools the skin as it evaporates. The latent heat required is “taken” from the surface, which is cooled [8]. If the skin of a hypothermic or cold-stressed patient is exposed to moisture, either following immersion in water or exposure to rain or snow, evaporation continues until either all the moisture is evaporated, or the surrounding air is 100% saturated.

International guidelines for treatment of accidental hypothermia recommend hypothermic patients to be wrapped in a protective “burrito” model with different layers that serves individual purposes [1]. One of the recommended layers is a vapor barrier, which is impermeable to moisture.

Evaporation is a major contributor to the heat loss in patients wearing wet clothing. Pre-hospital care for these patients should aim to minimize the effects of evaporation and increase rewarming rates, which may reduce hypothermia-related mortality and improve patient comfort. The purpose of the vapor barrier is to minimize evaporation from the patient’s clothes or skin. This is achieved by increasing the water vapor pressure inside the barrier, thereby reducing the driving gradient of evaporation, and limiting heat loss to a minimum. The volume of air, temperature, seal effectiveness/quality of the vapor barrier, and production (sweating) determine how quickly the air saturates. In addition, the vapor barrier protects any additional insulating material provided by rescuers by keeping it dry, thereby maintaining its insulating properties. The vapor barrier is generally recommended in the guidelines, but there is limited evidence regarding the specific effect of this layer, especially when combined with an active heat source [9]. Some evidence indicates that using a vapor barrier is beneficial, compared to using insulation alone, in patients with wet clothing in place [10]. The use of a vapor barrier is recommended by the Wilderness Medical Society Guidelines for out-of-hospital treatment of accidental hypothermia [11]. However, we are not aware of any studies demonstrating the isolated effect of using a vapor barrier.

We hypothesized that active external rewarming would accelerate evaporation inside the membrane. This would increase the speed of saturation of the air inside the vapor barrier which we hypothesized would lead to a reduction of evaporative heat loss. We also assumed that placing the vapor barrier closest to the patient and minimizing the volume of air saturated with water vapor would limit evaporative heat loss. Further, we speculated that thermal transfer from the heating device might be more effective if the volume of air inside the vapor barrier is saturated with water, because water vapor has a higher thermal coefficient than air. This study aimed to investigate the effect of using a vapor barrier as the inner layer in the recommended “burrito” model for wrapping hypothermic patients under field conditions on mean skin temperature and thermal comfort.

## Methods

### Ethics statements

The Norwegian Regional Ethics Committee for Medical and Health Research (2023/566,433 REK South-East C) and the Data Protection Officer of Haukeland University Hospital approved this study, which was registered with ClinicalTrials.gov (NCT 05779722). Written informed consent was obtained from all the participants.

### Study design and setting

This randomized, crossover field study of 16 healthy volunteers was conducted in Hemsedal, Norway during March 2023 to investigate the effect of using a vapor barrier as the inner layer in the recommended “burrito” model. This crossover design enabled all participants to undergo both control and intervention scenarios, serving as their own controls. The experimental setting was in a “snow cave” (7 m × 3 m × 1.80 m with approximately 1-m thick walls) built to serve as a field climate chamber to avoid wind and maintaining temperature and humidity as constant as possible during the experiments.

There was more than 2 h between the two sessions to allow sufficient restitution and avoid a carry-over effect. The snow cave had a room for four individual participants in each session, and eight sessions were conducted, adding up to 32 individual experiments. Each session was balanced, with two participants undergoing the intervention scenario and two participants undergoing the control scenario. Randomization by draw was performed on the day before the experiment, and randomization was blinded to all participants and during data processing.

### Selection of participants

Sixteen healthy volunteers (American Society of Anesthesiologists class 1) of both sexes older than 18 years of age were recruited. The exclusion criteria were history of smoking, previous abdominal surgery (due to the risk of complications from the ingested temperature telemetry pill), or acute sickness (fever or malaise) on the study date.

### Interventions

In both the control and intervention scenarios, participants were dressed in standardized cotton clothes soaked overnight in 1200 mL of water. At the start of the experiment, the participants were placed in a snow cave in the supine position on an insulating sleeping pad (Therm-a-Rest Z-lite, R-value 2.0, 2-cm foam) for 30 min to cool the skin. The cooling phase ended after 30 min, marking the start of the 60-minute rewarming phase. The participants were asked to stand up and lie back down in an open insulating model that had been positioned underneath them. The wrapping phase took approximately 5 min to complete.

**Fig. 1:**
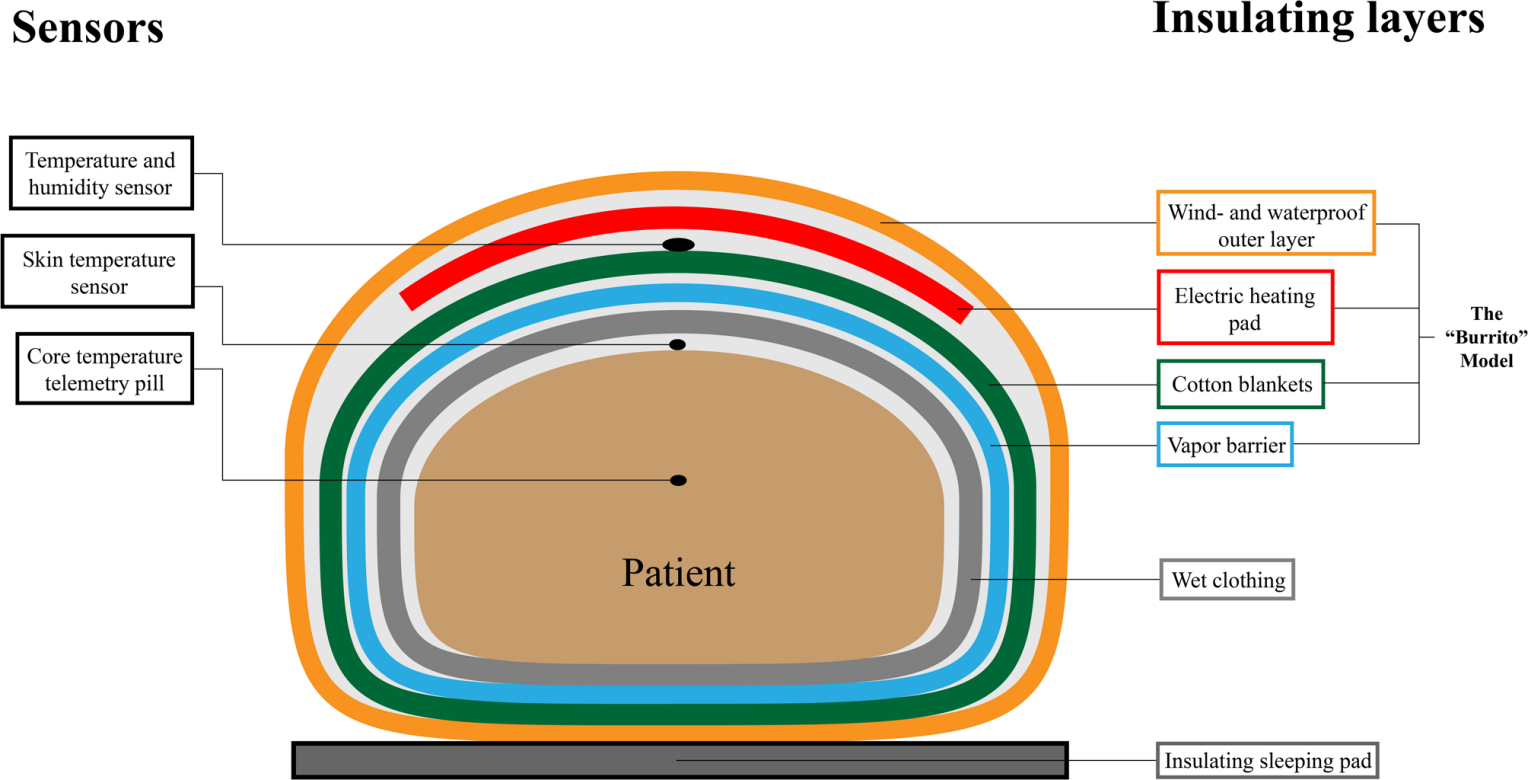
Illustration of “The Burrito model”. An illustrative cross-section of the different layers in our wrapping model, as well as the placement of the different sensors used in the experiment

Without removing the wet clothes, participants in the intervention scenario were wrapped with a watertight membrane (ASAP JONA 200™, 2 sheets taped together to achieve sufficient size to achieve a complete seal) as an innermost layer serving as a vapor barrier, to make the volume of air to be saturated with vapor as small as possible. Subsequently, overlapping woven cotton ambulance blankets (310 g/m^2^) were placed as the insulating layer, an electric resistive heating blanket (Geratherm Uniqueresc+, Geratherm Germany) on the highest setting (40 °C) as a source of active warming, and an insulated wind- and waterproof mountain quilt (Jerven Fjellduken Extreme Primaloft 170 g/m^2^, Jerven Norway) as an outer shell (Fig. 1).

The wrapping of the participants in the control scenario was identical, except for the vapor barrier. Each separate layer was applied simultaneously to each of the four participants during each run (for standardization purposes). This meant that all isolating blankets were applied simultaneously to all four participants after the vapor barriers had been applied to the two participants in the intervention scenario.

### Measurements and outcomes

The mean skin temperature, core body temperature, as well as temperature and humidity in the model were measured (Fig. 1). Shivering was measured subjectively by a modified questionnaire based on the Bedside Shivering Assessment Scale (BSAS) [12, 13]. Thermal comfort was evaluated using a questionnaire at baseline, 5 min, and then at 10-minute intervals during both the cooling and rewarming phases. The questionnaire can be found in the Additional files. Skin thermistors (iButton®, Maxim Integrated Products Inc.) were placed at seven predefined locations (the forehead, lower arm, hand, feet, lower leg, thigh, and abdomen), and the temperature was recorded every 30 s during the experiment. The mean skin temperature was calculated using a modified version of the weighted formula described by Hardy and Dubois [14, 15]. The core temperature was measured using an ingested thermal telemetry pill (eCelsius Performance, BodyCap Medical), which recorded the core temperature every 30 s during the experiment and was taken orally in the morning of the experiment. The temperature and humidity in the model were measured using a humidity and temperature data logger (OM-CP-MICRORHTEMP, Omega Engineering Inc.). Our primary outcome was the mean skin temperature, and our secondary outcomes were the core temperature, temperature and humidity in the model outside the vapor barrier, as well as the thermal comfort of our participants.

### Statistical analysis

Preliminary power analysis showed that assuming a minimal clinically relevant difference in mean skin temperature of 1 °C and a standard deviation of 0,8, we needed a sample size of 15 participants in each group to achieve a power of 0.9 with a two-sided t-test significance level of 0.05.

All temperature data are reported as mean ± standard deviation. Descriptive methods were used to characterize the samples. For the time-dependent outcomes (primary and all secondary outcomes), we fitted the analysis of covariance (ANCOVA) for each time point after baseline, i.e., the linear regression of the outcome value at the follow-up time point, depending on the treatment adjusted for the outcome at baseline. The time before wrapping was selected as the baseline. To address the crossover design, we added a random intercept per individual, and assumed carry-over effect to be zero. Missing data were excluded from the analysis of the time points at which they were missing. The general significance level was set at *p* < 0.05. The primary outcome consisted of a large number of highly correlated measurements. Therefore, multiple comparisons were not adjusted. Subjective thermal comfort was analyzed graphically. Data handling and computation were performed using R, version 4.3.1 (R Core Team) [16] and graphics in MATLAB 2023a (Mathworks Inc.)

## Results

A total of 16 research participants completed the study, and their baseline characteristics are presented in Table 1.

**Table 1:** Baseline characteristics. Baseline physical characteristics of the volunteer research participants

Randomization group 1st run, opposite for second run			
	All (*n* = 16)	Control (*n* = 8)	Intervention (*n* = 8)
Age (years)	27 [18, 59]	48 [18, 59]	24.5 [18, 47]
Sex (Female)	7 (44%)	5 (62%)	2 (25%)
Height (cm)	174 [155, 192]	172 [155, 180]	180 [160, 192]
Weight (kg)	72 [54, 120]	72 [63, 77]	74.5 [54, 120]
BMI	23.3 [19.4, 35.4]	23.2 [21, 32]	23.9 [19.4, 35.4]

The table shows the characteristics for run 1

### Mean skin temperature

**Fig. 2:**
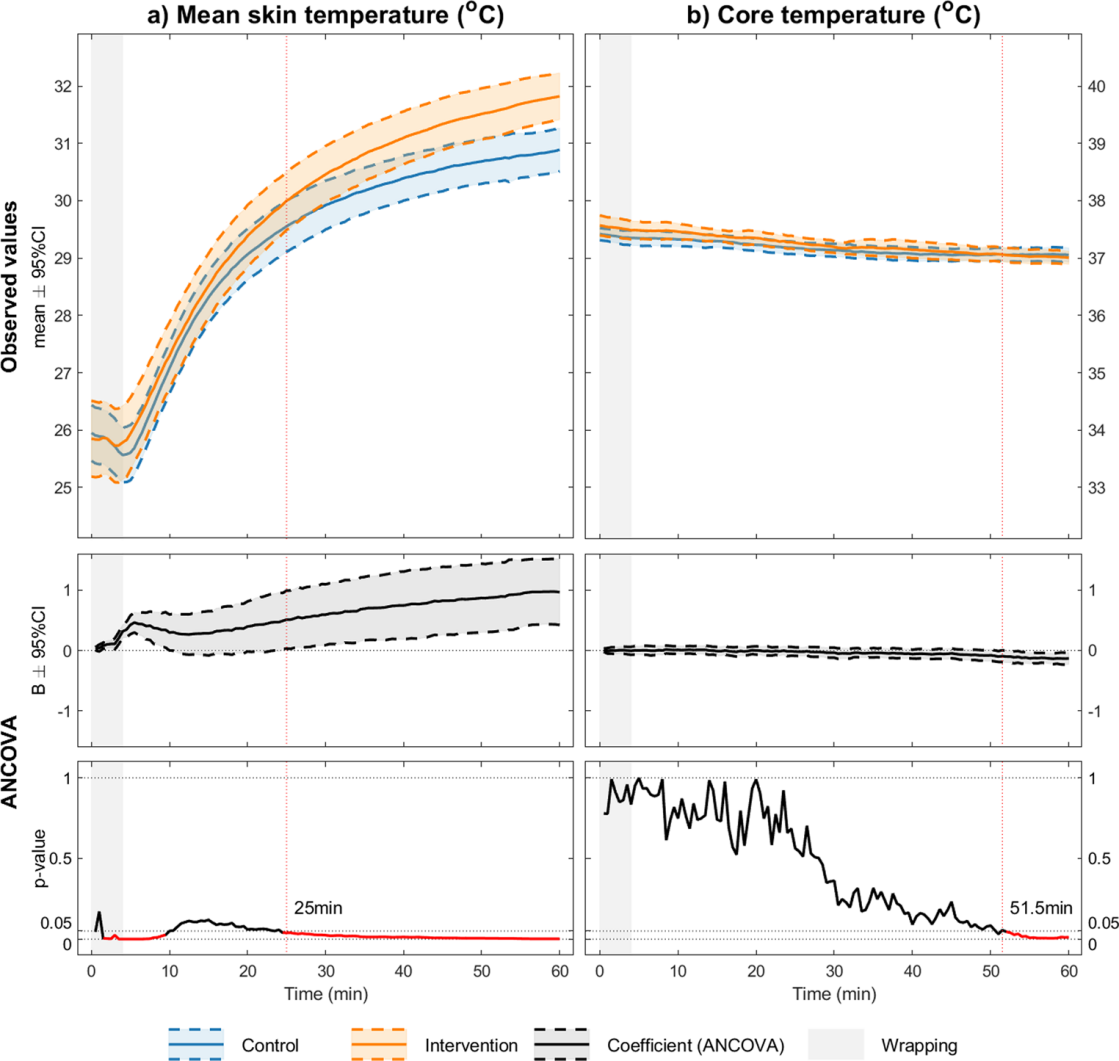
Temperature measurements. Graphical presentations of the measured mean skin temperatures (**a**) and core temperatures (**b**), as well as a 95% confidence interval and p-values for the difference between the control and intervention scenario

As shown in Fig. 2a, we observed a slightly higher temperature decrease at the end of the wrapping procedure than at the beginning, which remained significant for several minutes (2–10 min). After a subsequent period without significant differences between the intervention and control groups, the difference increased until the end of the rewarming period (60 min) and reached statistical significance at 25 min. The largest difference between the groups was 0.93 °C ± 0.59 °C, and the mean skin temperature was also different at the end of the experiment compared with that at the beginning (31.3 °C ± 0.4 °C versus [vs.] 30.3 °C ± 0.4 °C).

### Humidity and temperature outside the vapor barrier

**Fig. 3:**
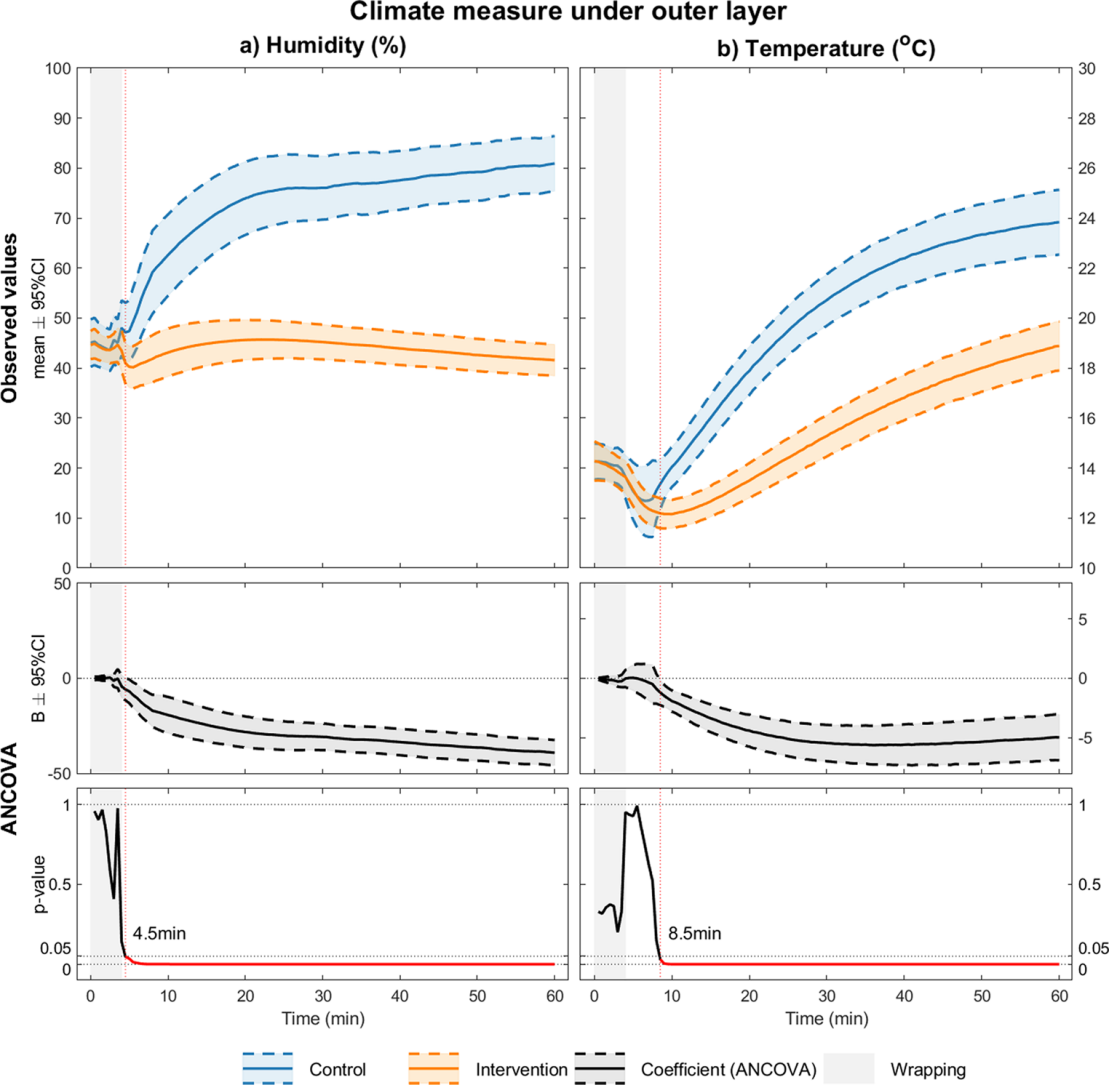
Measurements inside the model. Graphical presentations of the measured humidity levels (**a**, %) and temperatures (**b**, °C) underneath the outer layer in our model as well as a 95% confidence interval and p-values for the difference between the control and intervention scenario

Almost immediately after wrapping (4.5 min), we observed significantly higher humidity in the control group than in the intervention group. This coincided with an increase in temperature outside the vapor barrier in the control group (Fig. 3, after 8.5 min). The humidity increased rapidly to 75% after 20–25 min, and then slowed down. In the intervention group, the humidity remained stable until the end of the experiment. The largest difference in humidity between the groups was observed at the end of the experiment (81% ± 6% vs. 41% ± 3% at 60 min).

The temperature increased for both groups, with an increasing difference between the groups up to 30 min, and continued with a similar slope later. The largest humidity difference between the groups was observed at the end of the experiment 60 min later (81% ± 6% vs. 41% ± 3%), with a corresponding increase in temperature of approximately 6 °C (24 °C ± 1 °C vs. 18 °C ± 1 °C).

### Core temperature

There were no significant differences in core temperature between the groups during the experimental period (Fig. 2b). The control group experienced a decrease in core temperature of approximately 0.25 °C, whereas the intervention group showed a decreased of 0.4 °C over the total 90 min experimental period. In both groups, there was a continued decrease in the core temperature, even during the rewarming phase, but the slope decreased toward the end of the experiment.

### Subjective evaluations

**Fig. 4:**
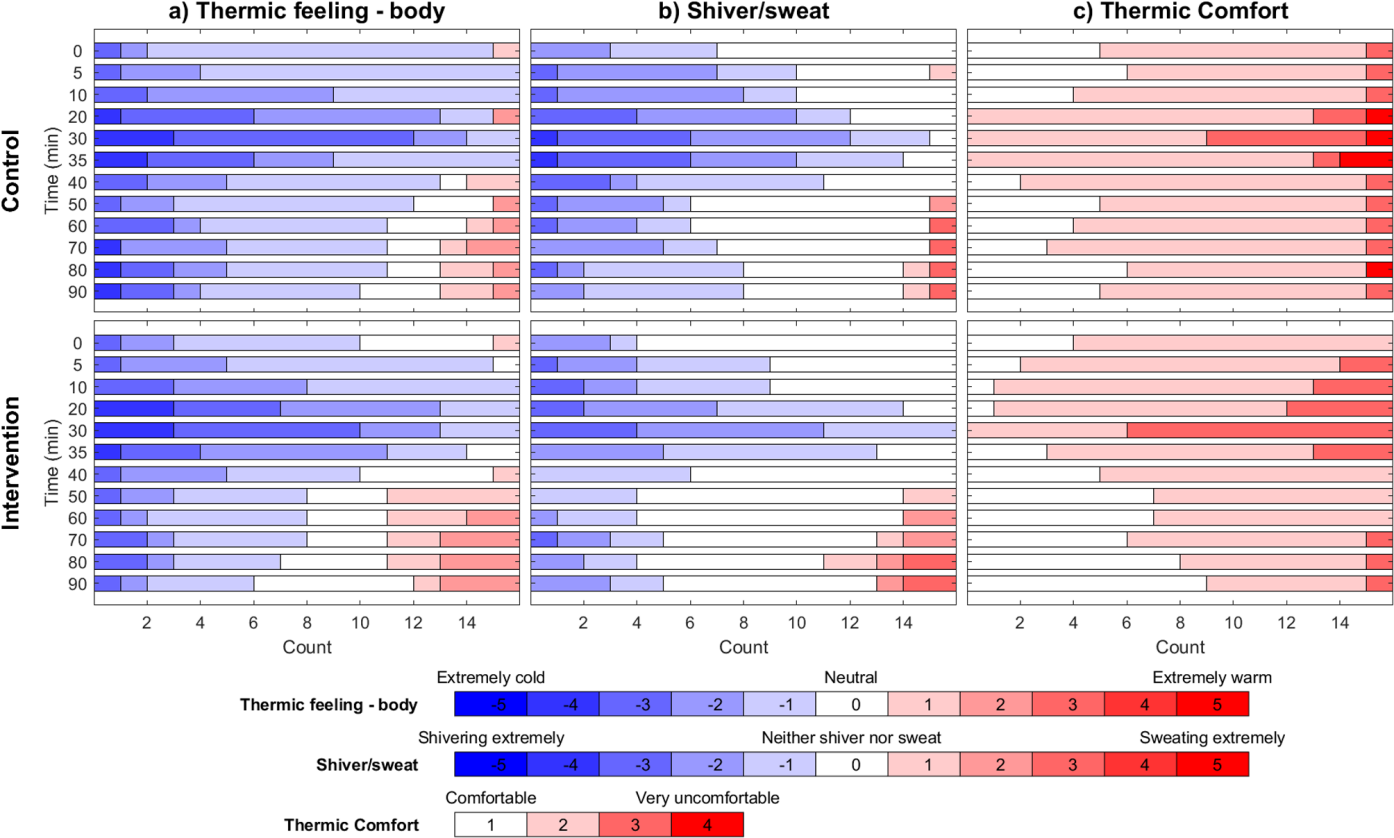
Subjective measurements of thermal sensation of the body, shivering and thermal comfort. Bar-plot presentation of our subjective measurements) for both the intervention and the control scenario. Thermal sensation of the body (**a**), subjective rating of shivering/sweating (**b**) and thermal comfort (**c**) are shown, along with an explanation of the values. Additional measurement results may be found in the supplementary material

As shown in Fig. 4, the intervention group generally reported feeling less cold than the control group; however, the p-values for this difference were not < 0.05.

There was also a clear trend toward higher thermal comfort in the intervention group than in the control group. (Fig. 4).

### Experimental procedure

All participants adhered to the protocol. The temperatures in the snow cave were stable on both study days (day 1: range, -3.0 °C–2.9 °C and mean, 1.8 °C; day 2: range, -2.3 °C–3.0 °C and mean, 0.8 °C). One set of core temperature measurements was missing (participant number 3, control scenario) owing to surprisingly short bowel transit time for the temperature pill. On one occasion, the temperature and humidity sensor was misplaced for the first 7 min of one experiment. Missing data were excluded from analysis.

## Discussion

The main finding of our study was that the mean skin temperature was higher in the intervention group than in the control group after 25 min, which lasted until the end of the experiment. The differences between the two groups continued to increase as the experiment progressed. The correlation between mean skin temperature and core temperature is non-linear and multifactorial, but the skin is the primary interface affected by external changes in temperature. The direct clinical relevance of the observed difference in mean skin temperature of 0.93 °C ± 0.59 is uncertain, and not the topic of this article. A priori, an increase in mean skin temperature will contribute to an increased core temperature rewarming rate in hypothermic patients after rescue or a reduced demand for metabolic heat production and oxygen consumption in patients at risk of accidental hypothermia.

We suspect that the observed initial difference between the two groups is attributable to the methodological decision of applying each corresponding layer simultaneously to all four participants present in the chamber simultaneously. The application of the vapor barrier meant that the wrapping of the participants in the control group was slightly delayed (approximately 60–90 s) compared with that in the intervention group, and we suspect that this delay is the main reason for the observed difference during the first 10 min. Therefore, we believe that this difference was of little relevance.

There was a clear difference in humidity levels between the intervention and control groups. Water that evaporated from the clothes appeared to be effectively contained within the vapor barrier, leading to lower levels of humidity outside the vapor barrier in the intervention group. In the control scenario, in which no vapor barrier was present, there was a rapid increase in humidity levels in the model during the first 20 min of the rewarming phase, followed by a period of slow but steady increase over the next 40 min of rewarming. Limiting evaporation inside the vapor barrier and maintaining latent heat close to the body may explain the higher rate of skin temperature increase observed in the intervention group.

Interestingly, our data showed a higher temperature between the insulating blankets and outer shell in the control group than in the intervention group. Some of this difference may be attributable to the isolating effects of the extra layer in the intervention group compared with that those in the control. However, this layer was extremely thin, and we suspect that the latent thermal energy present in the water vapor in the model was a substantial contributor to the temperature difference. The barrier used in the intervention group contained this vapor and, thereby, the thermal energy was closer to the patient, which may be the reason for the faster mean skin temperature rewarming rate in the intervention group.

Henriksson et al. found a higher rewarming rate with either a vapor barrier or wet clothing removal than wrapping a patient wearing wet clothing in an insulating material without a vapor barrier [10]. They found no difference between wet clothing removal and vapor barrier application. However, increasing the amount of insulating material provided similar effects as using a vapor barrier, perhaps because more material meant less vapor could escape. Hagen et al. found a higher rewarming rate when wet clothing was removed and the patient was placed in a vapor barrier compared with wrapping the patient in the vapor barrier with wet clothing still on [17].

Our core temperature measurements showed a slight decrease in core temperature for the entire 60-minute rewarming phase. There may have been slight heat stress for the participants during the application of the monitoring equipment before the study, followed by a slow return to baseline. This may also be attributed to a physiological phenomenon called afterdrop, in which the core temperature continues to decrease after a person is removed from a cold environment. The cold environment cools the more superficial tissues of the body; therefore, thermal diffusion from the core will continue after removal from the cold environment until an equilibrium between peripheral heat loss and central heat production is re-established. This is probably the reason for the delay in the core temperature increase from the surface rewarming observed in our study. We observed a slight decrease in the core temperature in both groups, and the temperatures would have been expected to return to baseline if the experimental period had been longer than 60 min. There was a slight difference between the groups after 50 min; the core temperature was slightly lower in the intervention group than in the control group. Our research participants were not hypothermic; therefore, it is possible that the active external heat source contributed to cutaneous vasodilation, causing increased blood flow to the cooled tissues, and resulting in a faster decrease in temperature and a shorter time until thermal equilibrium was reached. Thermoregulatory vasoconstriction is controlled centrally by the preoptic area of the hypothalamus. Vasoconstriction in a patient with accidental hypothermia continues despite peripheral heat stimuli by cutaneous rewarming for as long as the core temperature decreases [18]. There is no evidence supporting the historical claim that active external rewarming is dangerous; thus, most guidelines recommend active external rewarming as a treatment option [19–21]. The differences observed in our study were too small to be considered clinically relevant, and the study was not designed to detect differences in core temperatures between the groups. Therefore, we considered the relevance and importance of this finding to be negligible.

Although not completely blinded to the intervention, participants reported a higher degree of thermal comfort in the intervention group than those in the control group. Patient comfort is important in mountain rescue, as well as in healthcare. Thermal discomfort exacerbates pain and fear, and shivering is particularly uncomfortable [22, 23]. Providing active external rewarming may increase thermal comfort and the rewarming rate.

One study demonstrated the benefit of combining wet clothing removal before insulating the patient using a vapor barrier, insulating materials, and an outer shell [17]. Wet clothing removal will reduce the amount of thermal energy required to heat the volume of water encased inside the vapor barrier, but usually requires the patient to be undressed and exposed to the elements before being insulated in the wrap. A vapor barrier may still be useful if wet clothing is removed because snow or rain may enter the burrito model during wrapping or there may be residual moisture on the skin resulting is evaporative heat loss for the victim. Rapid, gentle and accurate application of the vapor barrier is essential for its effect. If the barrier is improperly placed, allowing continued vapor escape and heat loss from the model, it loses its function. Appropriate equipment, extensive training, and preparation are required for rescue services to achieve adequate insulation and patient protection without excessive movement of the patient.

Bubble wrap is frequently used as a vapor barrier material because it is believed to provide both a water-impermeable barrier and some level of insulation. However, the insulating properties of bubble wrap are limited, and the pack volume is large [9]. Consequently, rescue services should consider other waterproof materials with smaller volumes to achieve a similar performance.

### Limitations

In our study, we wrapped participants in the “burrito” model without removing their wet clothes. The removal of wet clothing may sometimes be advisable, and the combination of wet clothing removal and the use of a vapor barrier can be beneficial compared to only retaining water inside the model. The aim of our study was to evaluate the isolated effect of a vapor barrier, and not to compare the effectiveness of the two methods. Therefore, we chose not to remove the clothing to ensure a more consistent amount of water in the model. This approach achieves better standardization and increased internal validity than the other. However, this may be at the expense of external validity, as in a real-life scenario, the combination of wet clothing removal and the use of a vapor barrier could be beneficial. It is also important to note that in a real-life scenario, placing the heat source closer to the patient than demonstrated in our study would probably be advisable.

The vapor barrier used in our study (ASAP JONA 200) has a semi-permeable membrane (1213 g/m2/24 h) which may allow some vapor to escape over time. However, as shown in Fig. 3, the product worked as intended and we achieved a low and stable humidity outside the barrier. Since the objective of this study was to evaluate the general principle of limiting evaporation, and not to investigate specific products, we believe our results to be valid. It is also possible that the absorbing qualities of this blanket may have had an impact on the measured outcome.

Cotton blankets are not the optimal source of insulation in a mountain rescue scenario, and other materials such as wool, down, Primaloft or other would probably have yielded higher rewarming rates. However, we do not believe that it would significantly affect the results of our study as our primary goal was to evaluate the effect of eliminating evaporative heat loss, and the amount of insulation was identical in the two scenarios.

Another potential limitation of our study is that the outer layer of our model was impermeable to water vapor, creating an additional vapor barrier at the exterior of the model. This is in accordance with the guidelines for the care of patients under harsh conditions, where a wind and waterproof outer shell is recommended.

Lastly, an important limitation of our study is that water distribution in the garments may not have been complete. It is difficult to assess the impact this may have had, as uneven water distribution may have contributed to regional differences in skin temperature. However, there is no reason to believe that this affected one group of individuals differently from others.

## Conclusions

The use of a vapor barrier as the innermost layer effectively reduces evaporative heat loss when wrapping and isolating patients at risk of accidental hypothermia and leads to a faster mean skin rewarming rate.

### Electronic supplementary material

Below is the link to the electronic supplementary material.

Supplementary Material 1

Supplementary Material 2

Supplementary Material 3

Supplementary Material 4

Supplementary Material 5

Supplementary Material 6

Supplementary Material 7

Supplementary Material 8

Supplementary Material 9

## Data Availability

The datasets used and/or analyzed in the current study are available from the corresponding author upon reasonable request.

## Abbreviations

95% CI: 95% confidence interval
°C: Degree Celsius
T_core_: Body core temperature
T_skin_: Skin temperature
vs.: Versus
ANCOVA: Analysis of covariance
