# Supplementary figures and images for: Effect of a vapor barrier in combination with active external rewarming for cold-stressed patients in a prehospital setting: a randomized, crossover field study
Source: Scand J Trauma Resusc Emerg Med. 2024 Apr 25;32:35. doi: 10.1186/s13049-024-01204-2 (PMC11044347; doi:10.1186/s13049-024-01204-2)

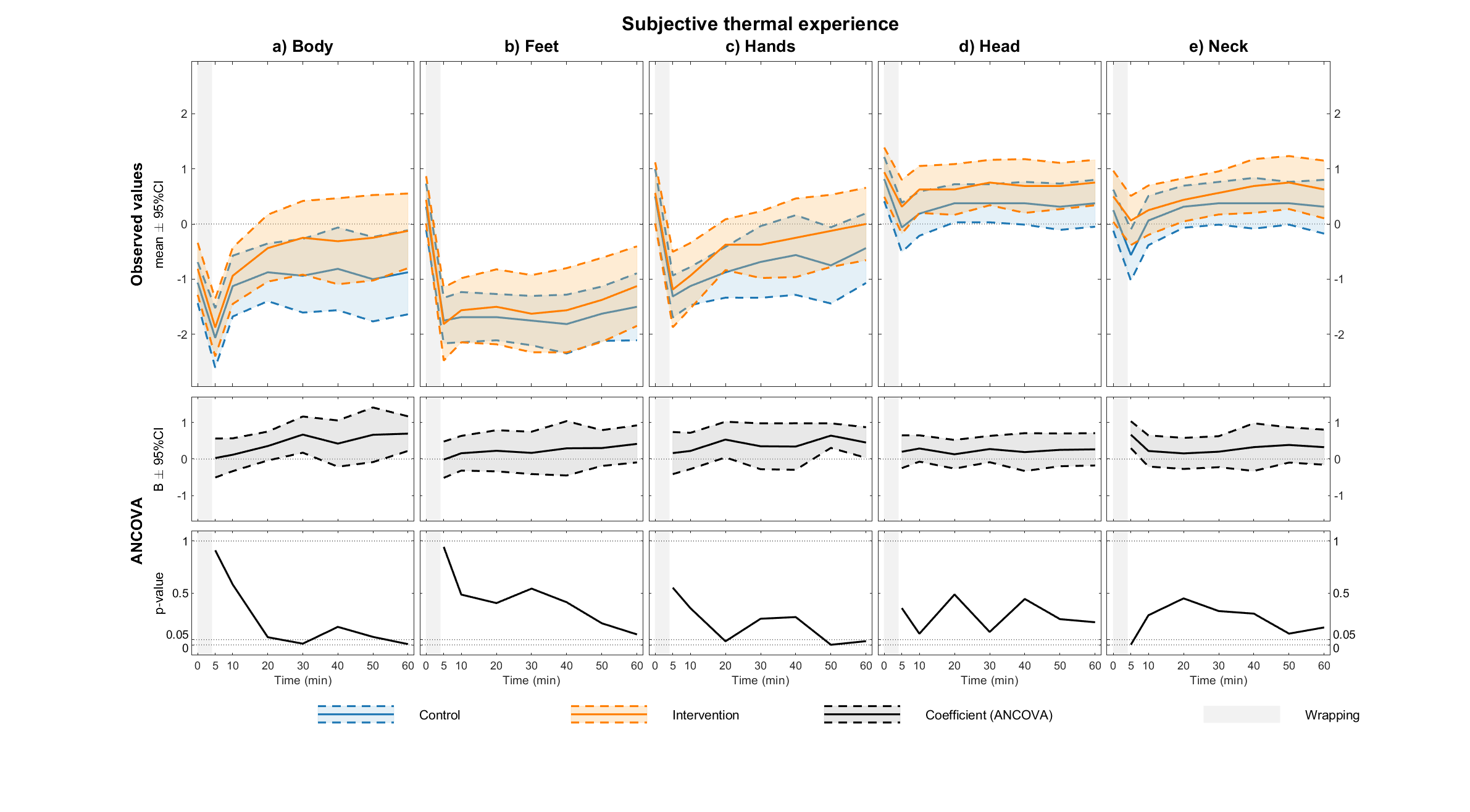

Supplement: Supplementary file 2 — Supplementary Material 2 [file 13049_2024_1204_MOESM2_ESM.tif]

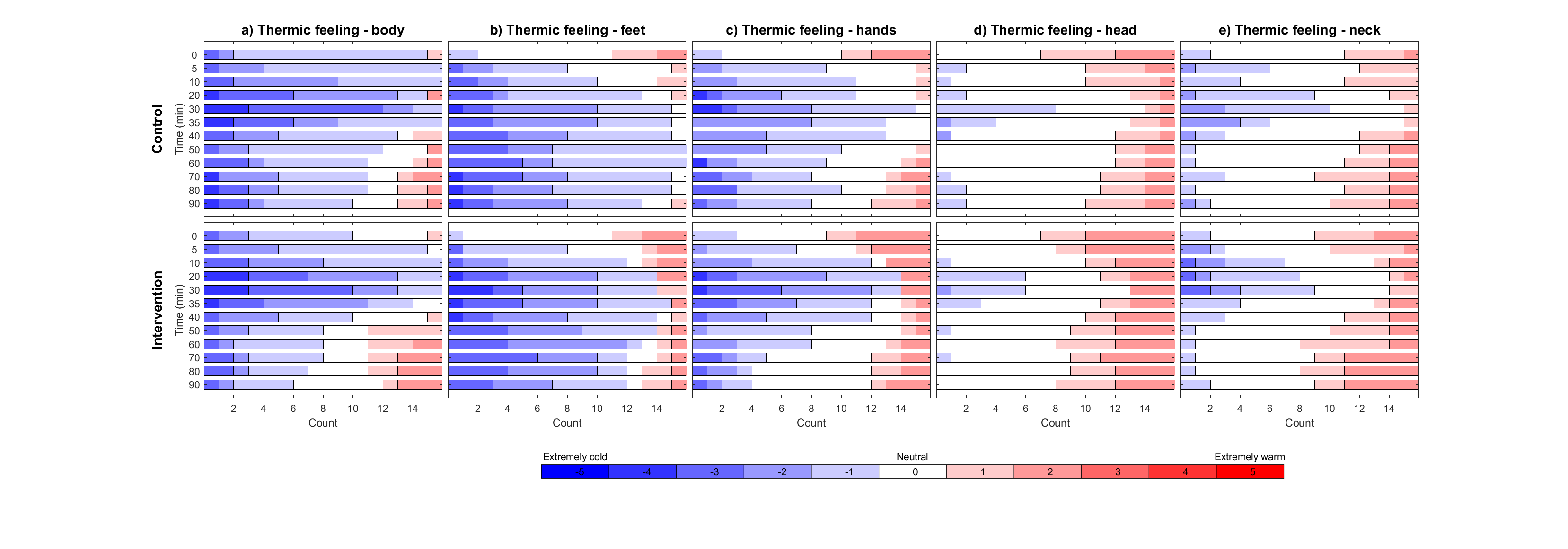

Supplement: Supplementary file 3 — Supplementary Material 3 [file 13049_2024_1204_MOESM3_ESM.tif]

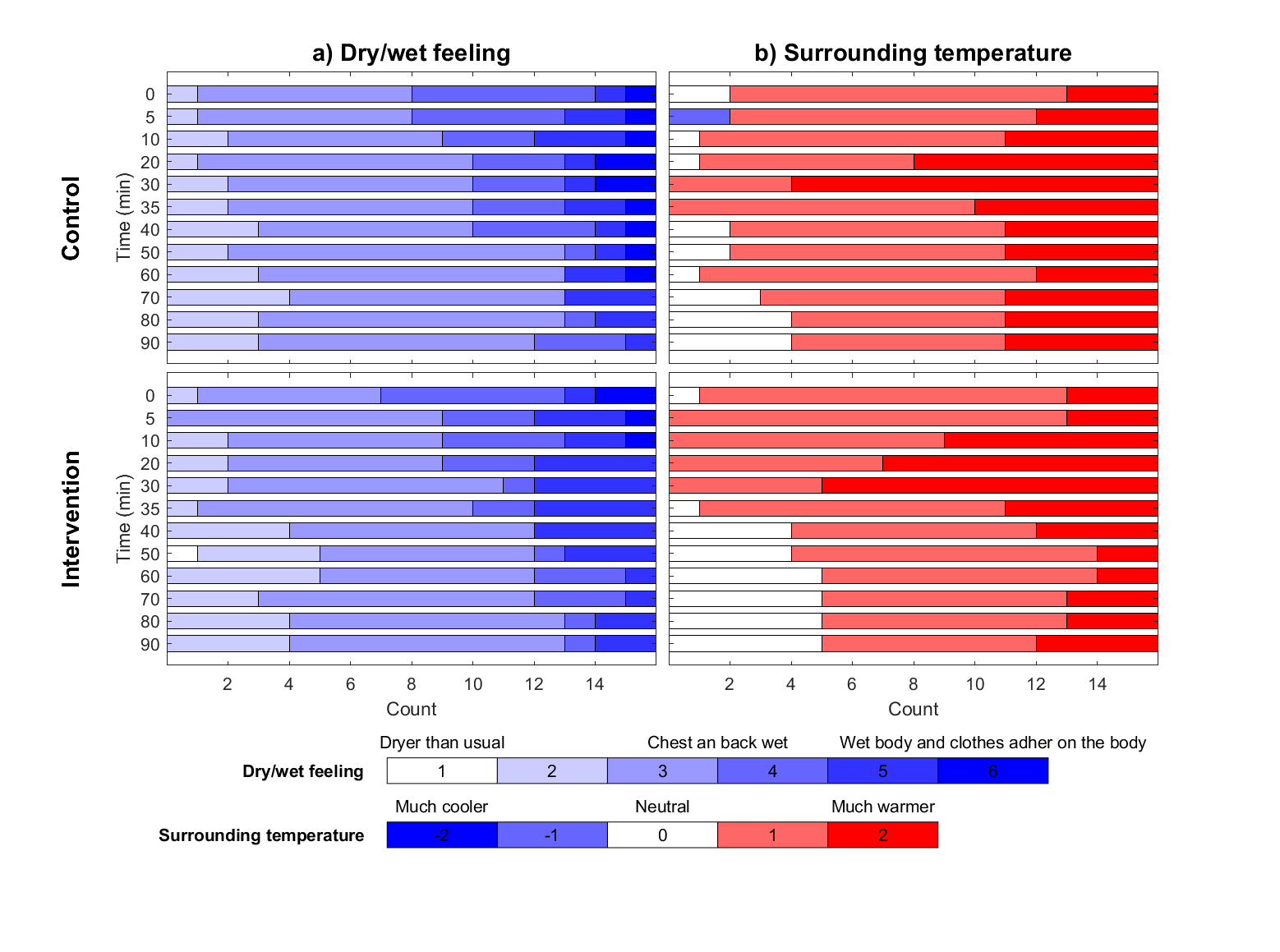

Supplement: Supplementary file 4 — Supplementary Material 4 [file 13049_2024_1204_MOESM4_ESM.tif]
