# Supplementary material for: Effect of a vapor barrier in combination with active external rewarming for cold-stressed patients in a prehospital setting: a randomized, crossover field study
Source: Scand J Trauma Resusc Emerg Med. 2024 Apr 25;32:35. doi: 10.1186/s13049-024-01204-2 (PMC11044347; doi:10.1186/s13049-024-01204-2)
Supplement: Supplementary file 5 — Supplementary Material 5 [file 13049_2024_1204_MOESM5_ESM.pdf]

MountainLab 2023 - Subjective evaluation questionnaire

Participant ID:	
Run:	

	Date	Time	Air temperature	Humidity
Start:				
End:				

		Before start	Cooling				Rewarming						
			5	10	20	30	5	10	20	30	40	50	60
1. 1.How is the thermal sensation of your	a) body												
	b) feet												
	c) hands												
	d) head												
	e) neck												
2. Shivering/ sweating													
3. How does your skin feel?													
4. 4.How would you prefer your surrounding temperature?													
5. 5.How do you feel with regards to thermal comfort?													

Følsomhet Intern (gul)
