# Supplementary material for: Effect of a vapor barrier in combination with active external rewarming for cold-stressed patients in a prehospital setting: a randomized, crossover field study
Source: Scand J Trauma Resusc Emerg Med. 2024 Apr 25;32:35. doi: 10.1186/s13049-024-01204-2 (PMC11044347; doi:10.1186/s13049-024-01204-2)
Supplement: Supplementary file 7 — Supplementary Material 7 [file 13049_2024_1204_MOESM7_ESM.pdf]

Subjektivt evalueringsskjema

1. **Hvordan føler du deg termisk i**
    a. Kroppen
    b. Føttene
    c. Henden
    d. Hodet
    e. Nakken

    -5 ekstremt kald
    -4 svært kald
    -3 kald
    -2 kjølig
    -1 litt kjølig
    0 nøytral
    1 litt varm
    2 varm
    3 het
    4 svært het
    5 ekstremt het

2. **Skjelving/svette. Du**

    -5 skjelver ekstremt av kulde
    -4 kraftig
    -3 moderat
    -2 litt
    -1 nesten ikke
    0 hverken skjelver eller svetter
    1 nesten ikke
    2 litt
    3 moderat
    4 kraftig
    5 svetter ekstremt

3. **Hvordan føles din hud**

    1 tørre enn normalt
    2 normalt tørr
    3 bryst og rygg lett våt
    4 bryst og rygg våt
    5 kroppen våt
    6 kroppen er vårt og tøyet kleber til Huden

4. **Hvordan vil du foretrekke omgivelsestemperatur?**

    -2 mye kjøligere
    -1 litt kjølig ere
    0 nøytral
    1 litt varmere
    2 mye varmere

5. **Hvordan føler du deg termisk tilpass?**

    1 komfortabel
    2 litt ukomfortabel
    3 ukomfortabel
    4 svært ukomfortabel
