# Supplementary material for: Effect of a vapor barrier in combination with active external rewarming for cold-stressed patients in a prehospital setting: a randomized, crossover field study
Source: Scand J Trauma Resusc Emerg Med. 2024 Apr 25;32:35. doi: 10.1186/s13049-024-01204-2 (PMC11044347; doi:10.1186/s13049-024-01204-2)
Supplement: Supplementary file 8 — Supplementary Material 8 [file 13049_2024_1204_MOESM8_ESM.pdf]

Mountainlab 2023 - subjektivt evalueringsskjema

Forsøksperson ID:	
Omgang:	

	Dato	Klokkeslett	Lufttemperatur	Luftfuktighet
Start:				
Slutt:				

		før start	Nedkjøling				Oppvarming						
			5	10	20	30	5	10	20	30	40	50	60
1. Hvordan føler du termisk	a) din kropp												
	b) dine føtter												
	c) dine hender												
	d) ditt hode												
	e) din nakke												
2. Skjelving/svette													
3. Hvordan føles din hud													
4. Hvordan vil du foretrekke omgivende temperatur													
5. Hvordan føler du deg termisk tilpass													

Klimakammer subjektivt evalueringsskjema, SINTEF, KS 78-05-02, v1.0, 2007-06-26, uten generelle spørsmål

Følsomhet Intern (gul)
