# Supplementary material for: Effect of a vapor barrier in combination with active external rewarming for cold-stressed patients in a prehospital setting: a randomized, crossover field study
Source: Scand J Trauma Resusc Emerg Med. 2024 Apr 25;32:35. doi: 10.1186/s13049-024-01204-2 (PMC11044347; doi:10.1186/s13049-024-01204-2)
Supplement: Supplementary file 9 — Supplementary Material 9 [file 13049_2024_1204_MOESM9_ESM.pdf]

Product data sheet – breathable film

Alfapac article number:	PA-0141
Thickness:	0,015 mm
Raw material:	GAIA Biodolomer® Vapour
**Test:**	WWTR (Water Vapour Transmission Rate)
**Test date:**	2018-07-12
**Test equipment:**	MOCON Permatran-W 101K
**Equip. calibration date:**	2018-06-12
**Test result:**	6 tests were made. Range 1183 – 1240 g.
**Average result:**	**1213 g** / sq.meter / 24 h / 37.8 degrees C / 760 mm Hg Barometer
**Alfapac prod. equipment:**	Alfapac extruder E06<br>BUR: 3,82<br>Screw designed for polyethylene-based blends with special mixing zone
**Product applications:**	Breathable laminating layer in non woven, and other structures, for hygiene and medical applications.

PA-0141 can be produced in various widhts, ranging from 500 – 1500 mm, depending on customer specification. Please contact us for further information.

Alfapac AB
Maskinvägen 2
694 60 Åsbro
Sweden

+46 (0)582 88 400
www.alfapac.se
